# Supplementary material for: In-Cell Determination of Lactate Dehydrogenase Activity in a Luminal Breast Cancer Model – ex vivo Investigation of Excised Xenograft Tumor Slices Using dDNP Hyperpolarized [1-13C]pyruvate
Source: Sensors (Basel). 2019 May 5;19(9):2089. doi: 10.3390/s19092089 (PMC6539471; doi:10.3390/s19092089)
Supplement: Supplementary file 1 [file sensors-19-02089-s001.pdf]

In-cell determination of lactate dehydrogenase activity in a luminal breast cancer model – *ex vivo* investigation of excised xenograft tumor slices using dDNP hyperpolarized [1-^{13}C]pyruvate

Supplementary Materials

Table S1- Summary of animals and tumor samples.

Experimental day / sample [a]	Mouse No.	Number of harvested tumors from each mouse	**Experimental Design 1** Perfusion Arrest and Non-Selective RF Pulses	**Experimental Design 2** Perfusion Arrest and Selective RF Pulses	**Experimental Design 3** Continuous Flow and Selective RF Pulses
	1	1			
1	2	1	2 injections		
	3	2			
	4	1			
2	5	1	1 injection [b]	3 injections [c]	
	6	1			
	7	1			
3	8	1		1 injection	
	9	1			
4	10	1		1 injection	
5	11	1			3 injections [d]

a - on each experimental day a single precision-cut tumor slices sample was used. This sample consisted of slices from all of the tumors that were harvested on that day.
b - Presented in Figures 3 and 4.
c - One of the injections is presented in Figures 5, 6a and 7a.
d - One of the injections is presented in Figures 6b and 7b.

Table S2- Summary of metabolic rate calculated for each injection

Experimental Day	Injection Number in Experimental Day	Experimental Design Used in the Injection	Calculated Metabolic Rate (nmole/nmole ATP in 1 min)	Calculated Metabolic Rate (µmole/g in 1 min)
1	1	1	5.92	5.09
	2	1	6.80	5.85
2	1[a]	1	5.44	4.68
	2[b]	2	0.92	0.79
	3	2	14.09	12.12
	4	2	2.45	2.11
3	1	2	1.44	1.24
4	1	2	0.82	0.71
5	1[c]	3	1.44	1.24
	2	3	1.30	1.12
	3	3	8.73	7.51

a - Presented in Figures 3 and 4.

b - Presented in Figures 5, 6a and 7a.

c - Presented in Figures 6b and 7b.

Determination of the [1-^{13}C]lactate and [1-^{13}C]pyruvate relative response to the selective excitation pulse

Note S1. Frequency selective pulse response.

We have previously demonstrated the observation of the production of hyperpolarized [1-^{13}C]lactate by frequency selective excitation pulses in another perfused tissue slices system (brain) [1]. In this technique, the metabolite of interest is fully sampled by each selective pulse, therefore, only newly synthetized metabolites were detected in the consecutive excitation, while the precursor ([1-^{13}C]pyruvate) is excited to a much lower degree. In order to quantify the LDH activity based on the signals of [1-^{13}C]lactate and [1-^{13}C]pyruvate, their relative response to the excitation pulse needs to be characterized. In this study we have used a 2.5 ms cardinal sine (sinc) pulse. The dependence of the signal on the pulse amplitude for on-resonance excitation and 214 Hz off-resonance (the frequency difference between lactate and pyruvate hydrate resonances at 5.8T) is shown in Figure S1. It can be seen that for the on-resonance excitation, the pulse response has the expected sine shape. However, for the off-resonance condition the maximum of excitation reaches a plateau at 17-36% (spectrometer's units). For this reason, we concluded that an effective "90°" excitation, or maximal excitation, can be achieved with a range of pulse amplitude therefore ensuring complete depolarization of the metabolite polarization while allowing a range of pyruvate-hydrate excitation levels (~90° – 270°).

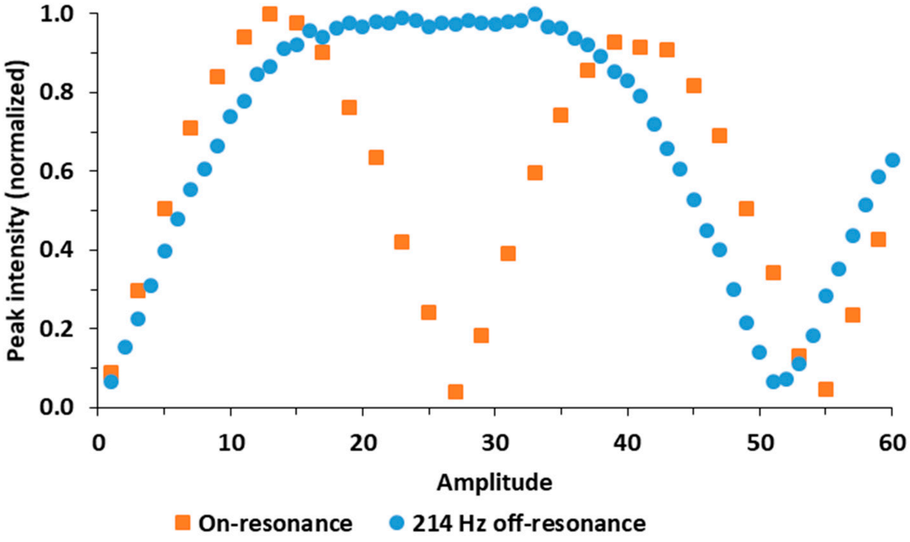

Figure S1. Dependence of the signal intensity on the selective pulse amplitude at on- and off-resonance conditions.

The on-resonance excitation (squares) shows a sine shape behavior (processed in magnitude mode). An off-resonance frequency of 214 Hz from the center, which corresponds to the distance from lactate to pyruvate hydrate at 5.8T (circles) shows non-sine shape behavior with a plateau of maximal excitation at 17-36% amplitude.

Note S2. Calibration of pulse profile.

For each experimental day, the amplitude of the frequency selective pulse was calibrated to yield a maximal dioxane signal on a standard sample of 40%/60% para-dioxane/deuterobenzene. The resulting amplitudes (21%, 24%, 26%) were all in the range that ensured maximal excitation of the lactate signal. However, the effect of these amplitudes changes on the overall pulse response profile was unknown. To this end, we carried out a series of off-resonance measurements on the same standard sample, where we applied the same pulse with 21%, 23%, and 26% amplitudes. As can be seen in Figure S2, the response of the pulse on-resonance varies greatly with the specific amplitude. The response of the pulse at ~214 Hz ([1-^{13}C]lactate to [1-^{13}C]pyruvate-hydrate) and at ~ -550 Hz ([1-^{13}C]pyruvate-hydrate to [1-^{13}C]pyruvate) was much less variable among the various amplitudes, yet not uniform. For this reason, a calibration of the pulse was performed on each experimental day.

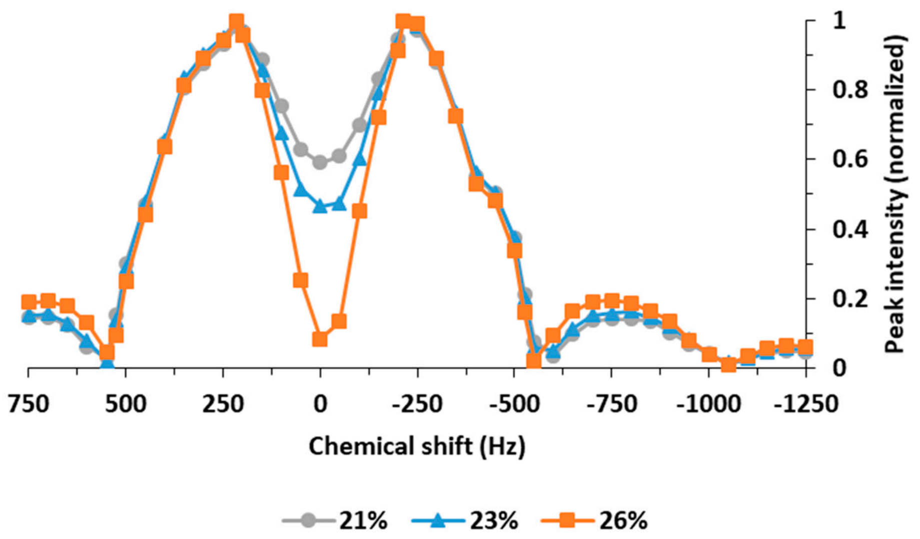

Figure S2. Calibration of pulse profile for obtaining the ratio of lactate-to-pyruvate excitation.
In this calibration we have used a repetition time of 70 s and 4 or 8 averages on the ^{13}C standard sample of para-dioxane and deuterobenzene described above. The pulse offset was changed to determine the pulse profile. Spectral processing was performed using a line broadening of 4 Hz on magnitude spectra. The maximum intensities of the para-dioxane middle signal are shown.
We chose to use the data acquired with an amplitude of 23% in order to calculate the pyruvate-to-lactate excitation ratio. For each injection the offset of lactate and pyruvate from the center of the pulse was measured and used in the calculation of the relative response of lactate and pyruvate to the selective excitation pulse (ϱ) such that $\varrho = \frac{pyruvate\ response}{lactate\ response}$. This ratio (ϱ) was then used to calculate the LDH activity rate as described in Eq. 3 in the text.

Note S3: Limitations

Validation of LDH activity or expression levels

We note that LDH activity in the tissue samples was not validated by another quantification assay. In the future we intend to validate this activity in tumor tissue slices and compare it to the activity in cultured cells. This will be done by monitoring the absorbance at 340 nm which is due to the concurrent production of NADH in the LDH reaction [2]. In addition, we intend to investigate whether our findings match the expression level of LDH as it has previously been shown that LDH-A is up-regulated in breast cancer and can be used as a prognostic biomarker [2,3]. Multiple assays have been used previously with regards to LDH activity in cancer such as immunohistochemistry [2,4], immunolocalization [5], ELISA [5], and western blotting [3,6-8].

Reference of activity to ATP level

The reference of the rates of [1-^{13}C]lactate production determined here to a tissue related variable was not trivial. Below is a discussion about the difficulty in assigning these rates to various tissue parameters. These explanations are also valid for any other hyperpolarized experiment done with tissue slices in an NMR tube as carried out previously in our lab [1,9].

a) Why not reference to tissue weight?

The entire tissue as represented by its weight in the NMR tube does not necessarily contribute to the measured production of hyperpolarized [1-^{13}C]lactate for the following reason:

1. Some of the tissue slices are located below or above the sensitive region of the NMR probe and therefore hyperpolarized [1-^{13}C]lactate produced in them would not be detected by the probe.
2. Because we have used 500 μm slices thickness, the center of each slice is likely not viable as the diffusion limits the adequate supply of nutrients and oxygen to a distance of about 125 μm. Thus, about one half of the slices weight likely contained non-viable tissue and therefore not metabolically active. In the future we intend to reduce the slice thickness to 350 μm in order to increase the slices viability.
3. Further presence of non-viable tissue can be induced during the slice preparation procedure.

b) Why not reference to tissue volume in the probe?

In some of the studies we have attempted to do this using the addition of the extracellular agent 3-aminopropylphosphonate [10] to the perfusion medium. Although this can add important information to aid quantification of the hyperpolarized [1-^{13}C]pyruvate signal, the respective volume occupied by the tissue still contains an unknown proportion of dead tissue (as explained above).

c) Why not reference to the protein content of the tissue?

The same rational that prohibits the use of tissue weight prohibits the use of the amount of protein in the tissue as a reference.

d) Why not extract the perfused tissue and compare to the metabolite content found in the tissue extract?

The same rational that prohibits the use of tissue weight prohibits the use of the extracted tissue metabolites as a reference. In addition, it is technically difficult to take out the perfused tissue out of the NMR tube used for perfusion fast enough such that freeze clamping technique can be employed. This will compromise the viability of the tissue at the time of freeze clamp and therefore the results of the extraction study.

e) The solution – reference to ATP level

The ATP level is observed in the same probe volume that is used for observing the hyperpolarized metabolites. Therefore, partial tissue considerations do not prohibit the quantification. In addition, the ATP level represents the viable and metabolically active cells, therefore the limitations imposed by partial viable tissue is removed. For this reason, we chose to use the ATP level of the tissue as a tissue based parameter to allow the quantification of the metabolic rates.

Reference

1. Harris, T.; Azar, A.; Sapir, G.; Gamliel, A.; Nardi-Schreiber, A.; Sosna, J.; Gomori, J.M.; Katz-Brull, R. Real-time ex-vivo measurement of brain metabolism using hyperpolarized [1-^{13}C]pyruvate. *Sci. Rep.* **2018**, *8*, 9564, doi:10.1038/s41598-018-27747-w.
2. Dong, T.; Liu, Z.; Xuan, Q.; Wang, Z.; Ma, W.; Zhang, Q. Tumor LDH-A expression and serum LDH status are two metabolic predictors for triple negative breast cancer brain metastasis. *Sci. Rep.* **2017**, *7*, 6069-6069, doi:10.1038/s41598-017-06378-7.
3. Huang, X.; Li, X.; Xie, X.; Ye, F.; Chen, B.; Song, C.; Tang, H.; Xie, X. High expressions of LDHA and AMPK as prognostic biomarkers for breast cancer. *Breast* **2016**, *30*, 39-46, doi:10.1016/j.breast.2016.08.014.
4. Koukourakis, M.I.; Giatromanolaki, A.; Sivridis, E. Lactate dehydrogenase isoenzymes 1 and 5: Differential expression by neoplastic and stromal cells in non-small cell lung cancer and other epithelial malignant tumors. *Tumour Biol.* **2003**, *24*, 199-202, doi:10.1159/000074430.
5. Chen, Y., Jr.; Mahieu, N.G.; Huang, X.; Singh, M.; Crawford, P.A.; Johnson, S.L.; Gross, R.W.; Schaefer, J.; Patti, G.J. Lactate metabolism is associated with mammalian mitochondria. *Nat. Chem. Biol.* **2016**, *12*, 937-943, doi:10.1038/nchembio.2172.
6. Javed, M.H.; Azimuddin, S.M.I.; Hussain, A.N.; Ahmed, A.; Ishaq, M. Purification and characterization of lactate dehydrogenase from Varanus liver. *Exp. Mol. Med.* **1997**, *29*, 25, doi:10.1038/emm.1997.4.
7. Fantin, V.R.; St-Pierre, J.; Leder, P. Attenuation of LDH-A expression uncovers a link between glycolysis, mitochondrial physiology, and tumor maintenance. *Cancer Cell* **2006**, *9*, 425-434, doi:10.1016/j.ccr.2006.04.023.
8. Lodi, A.; Woods, S.M.; Ronen, S.M. Treatment with the MEK inhibitor U0126 induces decreased hyperpolarized pyruvate to lactate conversion in breast, but not prostate, cancer cells. *NMR Biomed.* **2013**, *26*, 299-306, doi:10.1002/nbm.2848.
9. Lev-Cohain, N.; Sapir, G.; Harris, T.; Azar, A.; Gamliel, A.; Nardi-Schreiber, A.; Uppala, S.; Sosna, J.; Gomori, J.M.; Katz-Brull, R. Real-time ALT and LDH activities determined in viable precision-cut

mouse liver slices using hyperpolarized [1-^{13}C]pyruvate – implications for studies on biopsied liver tissues. *NMR Biomed.* **2019**, *32*, e4043, doi:10.1002/nbm.4043.

10. Gillies, R.J.; Liu, Z.; Bhujwalla, Z. P-31-MRS measurements of extracellular pH of tumors using 3-aminopropylphosphonate. *Am. J. Physiol.* **1994**, *267*, C195-C203.
